# Validation of Distinct Bladder Pain Phenotypes Utilizing the MAPP Research Network Cohort

**DOI:** 10.1007/s00192-024-05735-1

**Published:** 2024-02-01

**Authors:** Oluwarotimi Sewedo Nettey, Cindy Gu, Nicholas James Jackson, A. Lenore Ackerman

**Affiliations:** 1 Department of Urology, Baylor College of Medicine, Houston, TX 77030 USA; 2 Department of Urology, Division of Pelvic Medicine and Reconstructive Surgery, David Geffen School of Medicine at the University of California, Los Angeles, Box 951738, Los Angeles, CA 90095-1738 USA; 3 Department of Internal Medicine and Health Services Research, Geffen School of Medicine at the University of California, Los Angeles, Los Angeles, CA 90095 USA

**Keywords:** Symptom phenotypes, Bladder pain syndrome, Chronic pelvic pain syndrome, Interstitial cystitis, Machine learning analysis, MAPP research network cohort

## Abstract

**Introduction and Hypothesis:**

As interstitial cystitis/bladder pain syndrome (IC/BPS) likely represents multiple pathophysiologies, we sought to validate three clinical phenotypes of IC/BPS patients in a large, multi-center cohort using unsupervised machine learning (ML) analysis.

**Methods:**

Using the female Genitourinary Pain Index and O’Leary-Sant Indices, *k*-means unsupervised clustering was utilized to define symptomatic phenotypes in 130 premenopausal IC/BPS participants recruited through the Multidisciplinary Approach to the Study of Chronic Pelvic Pain (MAPP) research network. Patient-reported symptoms were directly compared between MAPP ML-derived phenotypic clusters to previously defined phenotypes from a single center (SC) cohort.

**Results:**

Unsupervised ML categorized IC/BPS participants into three phenotypes with distinct pain and urinary symptom patterns: myofascial pain, non-urologic pelvic pain, and bladder-specific pain. Defining characteristics included presence of myofascial pain or trigger points on examination for myofascial pain patients (*p* = 0.003) and bladder pain/burning for bladder-specific pain patients (*p* < 0.001). The three phenotypes were derived using only 11 features (fGUPI subscales and ICSI/ICPI items), in contrast to 49 items required previously. Despite substantial reduction in classification features, unsupervised ML independently generated similar symptomatic clusters in the MAPP cohort with equivalent symptomatic patterns and physical examination findings as the SC cohort.

**Conclusions:**

The reproducible identification of IC/BPS phenotypes, distinguishing bladder-specific pain from myofascial and genital pain, using independent ML analysis of a multicenter database suggests these phenotypes reflect true pathophysiologic differences in IC/BPS patients.

**Supplementary Information:**

The online version contains supplementary material available at 10.1007/s00192-024-05735-1.

## Introduction

Interstitial cystitis/bladder pain syndrome (IC/BPS) is a chronic, debilitating condition distinguished by pressure or pain perceived to be originating from the bladder [1]. Prevalence estimates suggest IC/BPS may affect as many as 7% of women [2]. While first publicly recorded in 1836 [3] as a syndrome of chronic frequency, urgency, dysuria, and pelvic pain, no objective consensus definition exists [1, 4–6].

The lack of clear diagnostic criteria for IC/BPS patients results in populations with heterogenous symptoms that often overlap with other urologic conditions, such as overactive bladder [7, 8]. Although IC/BPS likely consists of different subtypes of pain representing distinct pathophysiologies, IC/BPS is often diagnosed, studied, and treated as a single entity. Due to this heterogeneity, clinical trials and basic science studies face significant challenges advancing the understanding and treatment of IC/BPS [9, 10]. Better differentiation of distinct IC/BPS phenotypes is critical to improving our understanding and treatment of this condition.

While multiple IC/BPS phenotypes likely exist, only the presence of Hunner lesions on cystoscopy denotes a disease subgroup recommending a change in treatment [11, 12]. Other classification systems for patients without Hunner lesions have been proposed but remain underutilized due to reliance on extensive clinical information, detailed physical exams, or genetic/histopathologic results [13–15]. Our group recently evaluated symptomatic patterns in 145 women with IC/BPS without Hunner lesions [16]. Using unsupervised machine learning (ML) approaches, patients with bladder pain could be categorized into three distinct phenotypes using patient-reported symptomatic questionnaires alone. These three unique symptom clusters included: (1) myofascial pelvic pain (MFP) characterized by persistent pelvic discomfort, straining to void, urgency, frequency, and a sensation of incomplete emptying, (2) non-urologic pelvic pain (NUPP) exhibiting urethral and vaginal pain unrelated to voiding, and (3) bladder-specific pain symptoms (BPS) worsened with bladder filling and relieved by bladder emptying. These clusters showed varying responses to common IC/BPS therapies, aligning with their presumed etiologies. Notably, this classification system did not require physician assessment or examination, although it was confirmed on discriminate pelvic examination by a specialist, making it applicable to providers of any specialty or skill level. As this was a single-center cohort, however, it is unclear if these symptomatic clusters could be reliably reproduced in other populations.

The Multidisciplinary Approach to the Study of Chronic Pelvic Pain (MAPP) Research Network recruited a multi-center, national cohort of patients with IC/BPS to better understand the nature of the condition and its subgroups [17]. As clinicians have noted that patients with widespread pain respond differently than those with localized pelvic pain, three different phenotypes based on pain distribution have previously been proposed for the MAPP cohort: local pain, intermediate pain, or widespread pain, using detailed body mapping of painful regions [18]. We aimed to validate the findings of our prior single center pilot [16] in this larger, more geographically diverse patient cohort and determine the correlation of the pathophysiologic phenotype clusters defined in our pilot study with the widespreadness of pain described by body map phenotyping.

## Materials and Methods

### Participants

The MAPP (Multidisciplinary Approach to the Study of Chronic Pelvic Pain) Research Network is a multisite group of investigators supported by the National Institute of Diabetes and Digestive and Kidney Diseases (NIDDK), aiming to understand the etiology and natural history of IC/BPS. The current study uses data from the first observational cohort, which enrolled over 1039 participants at six clinical sites across the US from 2009 to 2014, including 233 women with IC/BPS for whom baseline phenotyping data could be obtained from the NIDDK repository (https://repository.niddk.nih.gov/home/) [17]. Participants provided written informed consent following institutional review board (IRB) approval at each recruiting site; the MAPP study design as well as the inclusion and exclusion criteria for this cohort have been previously described [17]. Local IRB exemption (IRB#21-000016) permitted the analysis of deidentified data.

Our initial pilot study prospectively enrolled premenopausal women (age < 45) suspected of having IC/BPS at their initial consultation with their provider; this single-center (SC) cohort characteristics and inclusion and exclusion criteria have been described previously. To ensure a population similar to the SC cohort, we excluded male patients (*n* = 191), women on opioids (*n* = 21), and women in the peri- and post-menopausal age range (age > 45, *n* = 82). Symptomatic clusters were generated from the remaining 130 women with IC/BPS in the MAPP cohort.

### Unsupervised ML Cluster Generation

Cluster definitions were based on patient-reported symptoms at baseline (at inclusion) on the pain, urinary, and quality-of-life (QoL) fGUPI subscales and the individual ICSI and ICPI measures. Prior to clustering, all scores were standardized using z scores. Using these measures, we applied a *k*-means clustering technique using Euclidian distance to classify the MAPP cohort into 2 to 20 homogeneous clusters. Selection of a 3-cluster solution was based on the information theoretic “jump method” approach of Sugar & James [19]. Patient cluster distribution was visualized by principal coordinate analysis with the Bray-Curtis dissimilarity measure.

### Cluster Analysis

Differences in patterns of co-existing urinary and pain complaints for the MAPP ML clusters were examined for measures not used for cluster generation: the American Urological Association Symptom Index (AUA-SI), the RAND Interstitial Cystitis Epidemiology Study Case Definition Questionnaire (RICE), and the Symptom and Health Care Utilization Questionnaire (SymQ). To determine if phenotypic ML clusters generated from the MAPP cohort aligned with those from the SC cohort, we compared patient-reported scores on symptom assessment instruments used in both studies (fGUPI, ICSI, and ICPI). MAPP ML clusters were also compared to body map phenotypes for the MAPP cohort, which were defined as previously reported [18].

### Cluster Stability and Validation

To ensure the stability of our cluster assignment, we used 500 bootstrap resamplings with replacement and identified the cluster assignments for each iteration. We computed the percent observed agreement (Rand Index) and percent overlap (Jaccard Coefficient), for which values over 0.7 indicate good cluster stability [20].

### Statistical Analysis

Differences in patients’ demographic and clinical characteristics between MAPP (or SC) cohort clusters or body map phenotypes were compared by using analysis of variance (or Kruskal–Wallis) and Pearson chi square (or Fisher exact), as appropriate. Post-hoc pairwise comparisons were assessed using Welch’s t-test (or Wilcoxon Rank Sum). Statistical significance was evaluated using a two-sided alpha level of 0.05. All analyses were conducted in Stata version 16.1 (StataCorp LLC; College Station, Texas).

## Results

### Baseline Characteristics of Cohorts

From the MAPP cohort, we identified 130 pre-menopausal women with IC/BPS who had complete baseline symptom assessment data and met inclusion and exclusion criteria. Demographics of the MAPP cohort are as follows: mean age 31.9 (7.4) years, 82% of the cohort identified as white, mean BMI was 24.6 (6.5) kg/m^2^, and 59% of patients had experienced symptoms for two or more years (Table 1). There were no significant differences in age or BMI between this cohort and the SC cohort; the SC cohort included 145 pre-menopausal women with a mean age of 31.8 (6.9) years and mean BMI of 25.4 (7.1) kg/m^2^, 73% of whom identified as white.

**Table 1:** Demographic and symptomatic features of the MAPP ML clusters

Cluster	MAPP C1-MFP	MAPP C3-BPS	MAPP-C2-NUPP	p
n	(31)	(52)	(47)	
Demographics				
Age (mean (SD))	31.05 (7.85)	31.20 (7.74)	31.96 (6.43)	0.827
Hispanic	3 (9.7)	4 (7.7)	4 (8.5)	0.952
Race category				0.922
White	24 (77.4)	45 (86.5)	40 (85.1)	
Black	2 (6.5)	1 (1.9)	2 (4.3)	
Asian	1 (3.2)	1 (1.9)	2 (4.3)	
Multi-race	1 (3.2)	1 (1.9)	2 (4.3)	
Other	3 (9.7)	4 ()	1 (2.1)	
Unknown	1 (3.2)	1 (1.9)	0 (0.0)	
Symptom duration in years (mean (SD))	1.65 (0.49)	1.52 (0.50)	1.66 (0.48)	0.311
BMI (mean (SD))	26.03 (8.73)	24.07 (5.36)	24.87 (6.29)	0.437
Anatomic findings/symptoms				
Pelvic floor musculature tenderness	23 (74.2)	23 (44.2)	17 (36.2)	0.003
Pelvic organ prolapse	0 (0.0)	1 (1.9)	1 (2.1)	0.085
Number of painful body sites (mean (SD))	10.29 (11.25)	7.08 (6.91)	5.51 (4.87)	0.027
Number of pelvic pain sites (mean (SD))	1.58 (1.18)	1.69 (1.02)	1.70 (1.12)	0.875
Number of sites outside of pelvic pain (mean (SD))	8.71 (10.57)	5.38 (6.39)	3.81 (4.51)	0.013
RICE features				
RICE subtype (%)				< 0.001
Painful filling	0 (0.0)	3 (5.8)	9 (19.1)	
Painful urgency	4 (12.9)	10 (19.2)	13 (27.7)	
Both	26 (83.9)	39 (75.0)	17 (36.2)	
Neither	1 (3.2)	0 (0.0)	8 (17.0)	
Pelvic pain (RICEq1, n (%))	30 (96.8)	51 (98.1)	43 (91.5)	0.271
Strong urge (RICEq2, n (%))	31 (100.0)	49 (94.2)	34 (72.3)	< 0.001
Urge with fear of incontinence (RICEq3, n (%))	1 (3.2)	0 (0.0)	4 (11.8)	0.034
Pain with bladder filling (RICEq4, n (%))				0.026
Gets worse with filling	25 (80.6)	43 (82.7)	26 (55.3)	
Gets better with filling	1 (3.2)	1 (1.9)	2 (4.3)	
Stays the same with filling	4 (12.9)	9 (17.3)	19 (40.4)	
Daytime frequency (RICEq5, mean (SD))	14.13 (5.65)	12.58 (6.78)	7.83 (2.94)	< 0.001
AUA Symptom Index				
Incomplete emptying (AUA-SIq1, mean (SD))	3.81 (1.47)	3.38 (1.52)	1.51 (1.18)	< 0.001
Urinary frequency (AUA-SIq2, mean (SD))	4.53 (1.14)	4.02 (1.02)	1.87 (1.10)	< 0.001
Intermittency (AUA-SIq3, mean (SD))	3.19 (1.74)	2.58 (1.65)	1.34 (1.48)	< 0.001
Urinary urgency (AUA-SIq4, mean (SD))	4.06 (1.24)	2.71 (1.39)	1.70 (1.61)	< 0.001
Slow stream (AUA-SIq5, mean (SD))	3.03 (1.74)	2.06 (1.69)	1.13 (1.28)	< 0.001
Strain to void (AUA-SIq6, mean (SD))	2.84 (1.86)	2.31 (1.82)	0.57 (1.08)	< 0.001
Nocturia (AUA-SIq7, mean (SD))	3.42 (1.12)	1.88 (1.06)	1.15 (0.81)	< 0.001
* AUA-SI total score (mean (SD))*	24.84 (6.92)	18.94 (5.96)	9.28 (5.52)	< 0.001
Symptom and Health Care Utilization Questionnaire (SymQ)				
Pain/pressure severity (SymQ1, mean (SD))	7.10 (1.72)	5.73 (1.62)	3.96 (1.82)	< 0.001
Urgency severity (SymQ2, mean (SD))	7.45 (1.31)	5.85 (1.99)	3.77 (2.21)	< 0.001
Urinary frequency (SymQ3, mean (SD))	7.42 (1.48)	5.56 (1.92)	3.17 (2.14)	< 0.001
Daytime frequency (SymQ4, mean (SD))	3.33 (0.71)	2.77 (0.90)	1.79 (0.72)	< 0.001
Urologic/Pain symptom severity (SymQ5, mean (SD))	7.61 (1.67)	5.83 (1.69)	3.83 (2.06)	< 0.001
Persistent pain not urologic/pelvic (SymQ6, mean (SD))	5.03 (2.75)	3.46 (2.75)	2.81 (2.47)	0.002
Mood (SymQ7, mean (SD))	5.68 (2.40)	4.42 (1.71)	4.13 (1.96)	0.003

Geometric mean scores and standard deviations (mean (SD)) or total numbers of participants endorsing symptoms with percentages affected (n (%)) are shown as indicated. RICE, RAND Interstitial Cystitis Epidemiology Study Case Definition Questionnaire; AUA-SI, American Urological Association Symptom Index; SymQ, Symptom and Health Care Utilization Questionnaire

### Unsupervised Clustering of MAPP Dataset

**Fig. 1:**
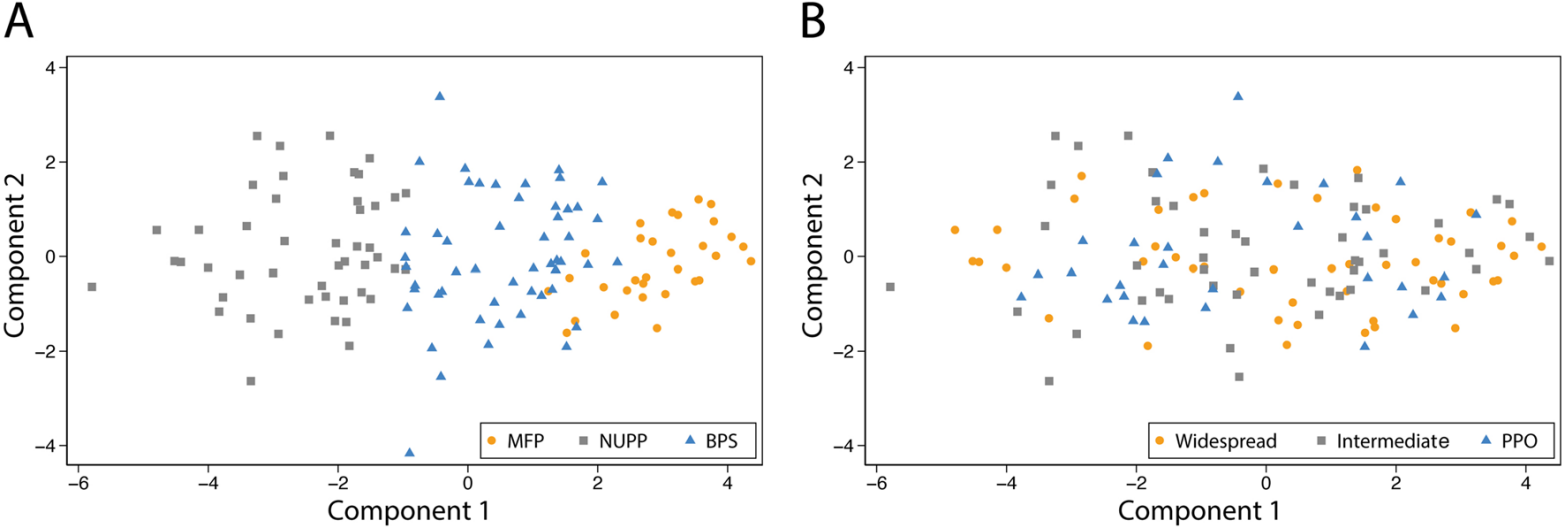
Principal component analysis of MAPP cohorts. When classified using the ML algorithm (**A**), MAPP clusters demonstrated good separation. In contrast, when patients were classified into groups based on body map patterns (**B**), no distinction between groups could be visualized

We used *k*-means clustering, an unsupervised ML approach, to identify distinct phenotypes in the MAPP cohort based on patient-reported fGUPI subscores and ICSI/ICPI features. We identified a three-cluster solution that assigned 31 participants to group 1, 47 to group 2, and 52 to group 3. The meaningful separation of these groups was visually confirmed by principal coordinate analysis incorporating symptomatic assessment measures for urinary and pain symptoms using Bray–Curtis dissimilarity measures (Fig. 1A). In contrast, when these patients were classified using the previously described phenotyping schema based on spatial distribution of pain (body map subgroups), the combined features of the derived clusters overlapped extensively (Fig. 1B).

To examine the stability of our cluster determinations, we computed the percent observed agreement (Rand Index) and percent overlap (Jaccard Coefficient) for each cluster for which a value greater than 0.7 indicates good cluster stability. Rand indices for all three groups ranged from 0.74–0.76 (Supplemental Table 2), demonstrating high percent observed agreement of the bootstrapped samples. The Jaccard coefficients were 0.51 for MAPP C1, 0.64 for MAPP C2, and 0.42 for MAPP C3.

## Baseline Cluster Characteristics of the MAPP Cohort

**Fig. 2:**
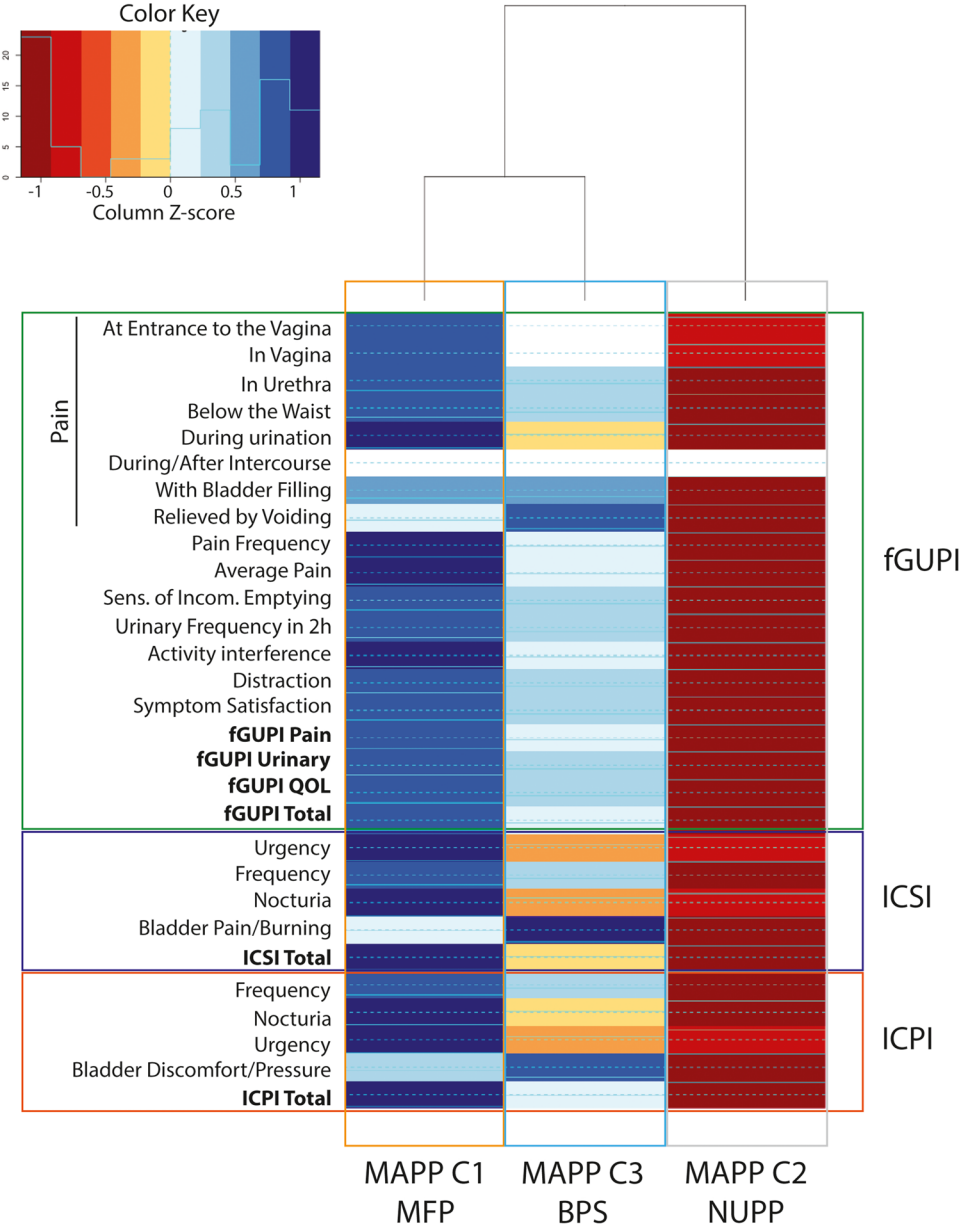
Differences in Cluster Determinants between Unsupervised ML Clusters for the MAPP Cohort. Clinical urinary and pain features for each cluster were expressed as Z scores to provide a normalized distribution relative to the mean of the overall population regardless of individual item scale. These were then expressed as a heat map for each individual question score and subscale total to provide a visual representation of features uniquely associated with each phenotypic group

Examination of scores on the patient-reported symptomatic questionnaires used for ML clustering revealed significant differences in the patterns of associated symptoms for painful and urinary symptoms (Table 1). Overall, participants in the first cluster (MAPP C1) had the greatest severity of lower urinary tract symptoms (fGUPI urinary scale, individual ICPI/ICSI questions), genitourinary pain (fGUPI pain scale), and symptomatic bother (GUPI QoL impact score) (Fig. 2, Supplemental Table 1). However, assessments evaluating urinary urgency (ICSI1, ICPI3), nocturia (ICSI3, ICPI2), and dysuria (fGUPIq2a) were particularly prominent in this cluster.

The second cluster (MAPP C2) exhibited pelvic pain unrelated to the voiding cycle, with the lowest scores for bladder pain/discomfort (ICSI4, ICPI4), pain related to micturition (fGUPIq2c, d), and average pain severity (fGUPIq4). While this group demonstrated the lowest symptom severity and impact on quality of life (Fig. 2, Supplemental Table 1), their symptoms scores for pain at the vaginal introitus (fGUPIq1a) and vagina (fGUPIq1b), pain below the waist (fGUPIq1d), and pain with sexual intercourse (fGUPIq2b) were not significantly different from the other clusters.

The third group (MAPP C3) overall had milder symptoms than MAPP C1 but was distinguished by bladder-specific pain (ICSI4, ICPI4) aggravated by bladder filling (fGUPIq2c) and relieved by emptying (fGUPIq2d). Despite this relationship of symptoms to the voiding cycle, MAPP C3 exhibited lower levels of frequency (ICSI2, ICPI1) than MAPP C1. MAPP C3 tended to have intermediate symptom severities and bother levels when compared to MAPP C1 and MAPP C2 (Fig. 2).

## Overlap of Cluster Phenotypes between MAPP and SC Dataset

**Fig. 3:**
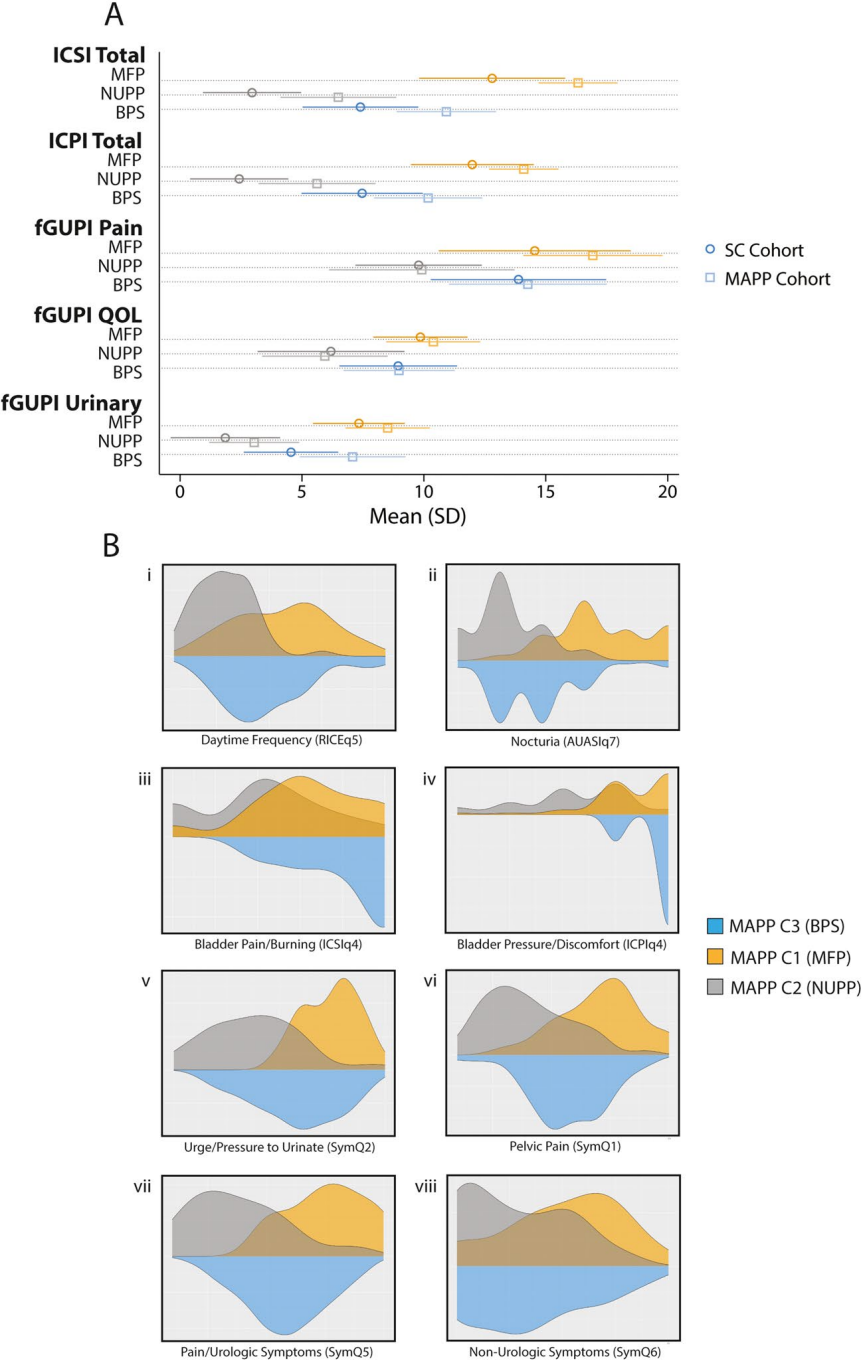
Symptomatic cluster features. **A** *Comparison of ICSI/ICPI/GUPI scores between MAPP and SC clusters.* While the MAPP cohort tended to demonstrate more severe symptoms, there was substantial overlap in symptoms scores and relative severities for all domains between the two independent cohorts. **B** *Scaled patient scores on a range of painful and urinary complaints for the MAPP cohort clusters.* Mirrored density plots display the differences in the distributions of patient responses for each cluster for (i) urinary frequency during the day; (ii) nocturia; (iii) bladder pain; (iv) bladder pain, burning, pressure, or discomfort; (v) an urge or pressure to urinate; (vi) generalized pelvic pain; combined pelvic pain and urologic complaints (vii); and non-urologic symptoms (viii)

To determine the symptomatic overlap of the ML-derived MAPP cohort clusters with the previously defined bladder pain phenotypes in the SC cohort, we compared patient-reported symptoms for the overlapping measures of the fGUPI, ICSI, and ICPI. While the MAPP cohort exhibited more severe symptoms overall, the relative patterns were similar on all measures used for clustering (Fig. 3A), demonstrating good concordance between the MAPP and SC clusters. MAPP C1 (C1-MFP) aligned with the myofascial pain cluster (SC-MFP) in the SC cohort, MAPP C2 (C2-NUPP) exhibited similar scores as the non-urological pelvic pain cluster (SC-NUPP), and MAPP C3 (C3-BPS) shared key features with the bladder-specific pain symptoms group (SC-BPS).

As the SC-MFP cluster had been defined by the presence of myofascial pain and trigger points on physical exam, we examined the presence of pelvic floor musculature tenderness across the MAPP clusters (Table 1). The C1-MFP cluster had a significantly higher proportion of participants with pelvic floor musculature tenderness (74%, *p* = 0.003) in comparison to the C2-NUPP (36%) and C3-BPS (44%) clusters, consistent with a pelvic floor etiology to this cluster’s pain.

## Distinct, Reproducible Symptomatic Patterns for ML Clusters by Urologic Phenotyping

To validate the consistency of each ML cluster’s symptomatic patterns, we also assessed urinary and pain symptoms on symptomatic measures not utilized for clustering (Fig. 3B). The subjective severity of frequency (AUA-SIq2) was similar between the C1-MFP (4.5 ± 1.1) and C3-BPS (4.0 ± 1.0) clusters, which were both significantly higher than C2-NUPP (1.9 ± 1.1, *p* < 0.001). However, even though the C3-BPS group complained of pain related to the voiding cycle, when participants were asked to quantify daytime frequency (RICEq5), the C1-MFP cluster exhibited the highest number of daytime voids (14.1 ± 5.7, *p* < 0.001, Fig. 3Bi), suggesting this group exhibited a more persistent need to urinate than C3-BPS (12.6 ± 6.8) or C2-NUPP (7.8 ± 2.9). This difference was more profound for nocturia (AUA-SIq7); all participants in C1-MFP exhibited nighttime urination, averaging more than three episodes of nocturia per night (3.4 ± 1.1), in contrast to C3-BPS (1.9 ± 1.1, *p* < 0.001) or C2-NUPP (1.2 ± 0.8, *p* < 0.001). The severity of an “urge or pressure to urinate" (SymQ2) was also most severe in the C1-MFP cluster (7.5 ± 1.3), with intermediate levels in C3-BPS (5.9 ± 2.0, *p* < 0.001) and the lowest levels in C2-NUPP (3.8 ± 2.2, *p* < 0.001).

As previously observed in the SC cohort, significant differences were observed for the magnitude of bladder pain according to question wording (Fig. 3Biii–vi). When specifically asked about bladder pain or burning (ICSIq4), only C3-BPS exhibited high severity scores (4.1 ± 1.1), significantly elevated over C1-MFP (3.2 ± 1.4, *p* = 0.001) and C2-NUPP (2.1 ± 1.5, *p* < 0.001) scores. When the question included bladder pressure and discomfort as well (ICPIq4), however, C2 scores remained lower (2.2 ± 1.2, *p* < 0.001), but C1-MFP scores (3.35 ± 0.9) were closer to C3-BPS (3.8 ± 1.5, *p* = 0.005). In almost all participants, the urge to void (RICEq3) was prompted by pain or discomfort; while a small number of participants did endorse urgency related to a fear of incontinence in the C1-MFP (3%) and C2-NUPP (12%), this complaint was absent from the C3-BPS cluster (*p* = 0.03); those patients all endorsed painful urge.

In general, the C1-MFP exhibited the highest severity scores on a range of painful and urologic symptoms (represented by SymQ5, Fig. 3Bvii; 7.6 ± 1.7), with C2-NUPP as the least severe (3.8 ± 2.1, *p* < 0.001) and C3-BPS displaying an intermediate phenotype (5.8 ± 1.7, *p* < 0.001). When asked about the severity of other sources of persistent pain that were neither urologic or pelvic in origin on an 11-pt Likert scale (SymQ6, Fig. 3Bviii), the C1-MFP cluster endorsed elevated levels (5.0 ± 2.8) of pain unrelated to the bladder, in comparison to C3-BPS (3.5 ± 2.8, *p* = 0.014) and C2-NUPP (2.8 ± 2.5, *p* < 0.001).

## Patterns of Bodily Pain By Cluster

**Fig. 4:**
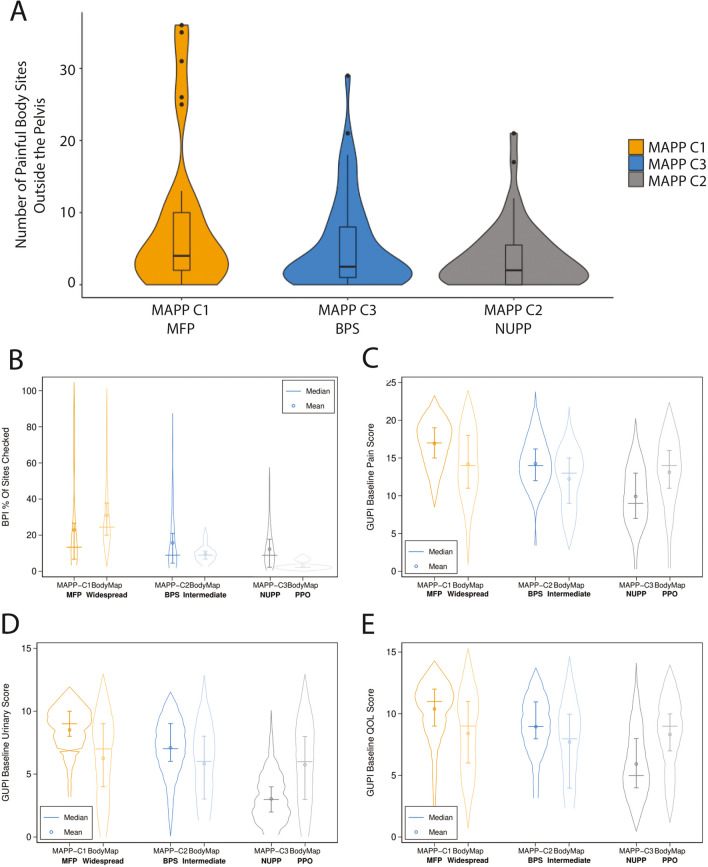
Comparison of MAPP ML clusters and body map phenotypes***.*** **A** The number of painful body sites outside the pelvis is shown in a violin plot with the median indicated by a bold line, and the box indicating the first and third quartiles. **B**–**E** Comparison of the distribution of responses on the total number of body sites with pain (**B**), fGUPI Pain Subscale (**C**), fGUPI Urinary Subscale (**D**), and fGUPI Quality of Life Subscale (**E**) for the MAPP ML clusters and body map (BM) phenotypes

**Fig. 5:**
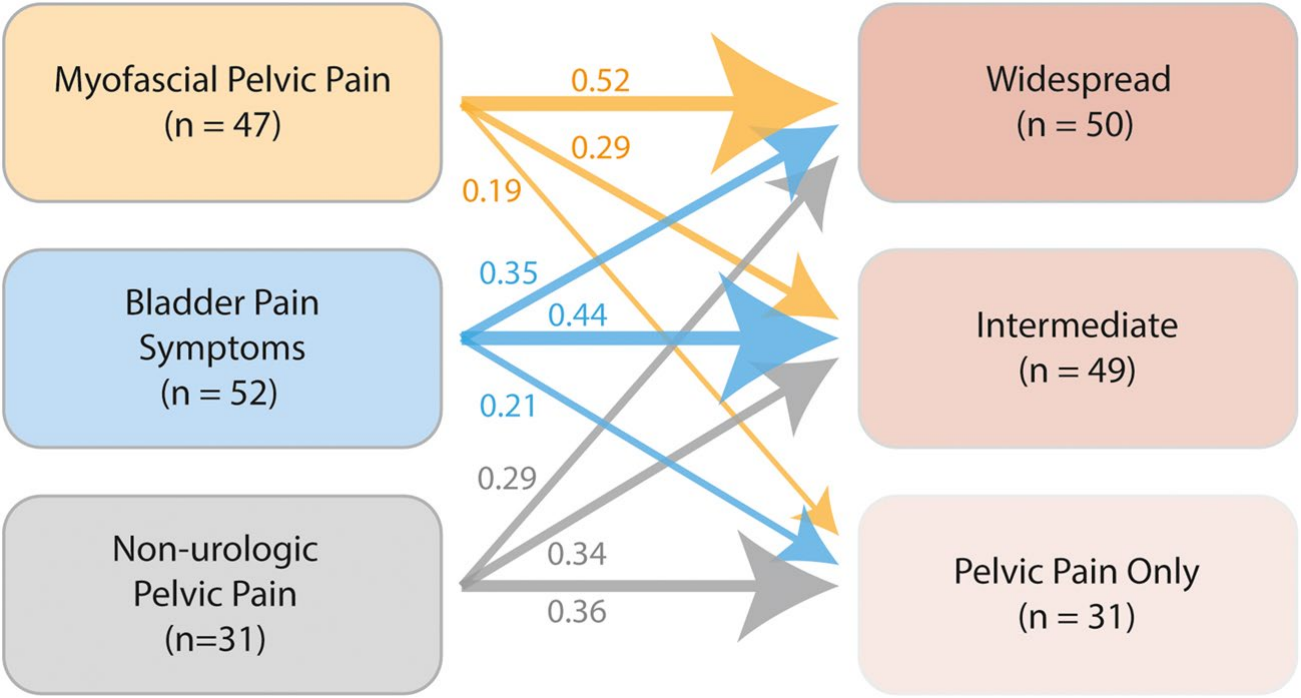
Overlap in ML catergorization of MAPP patients with the body map phenotyping. The size of the arrow is proportional to the amount of the ML cluster population which categorized to the indicated body map phenotype, which is also indicated in the decimal beside each arrow that denotes the proportion of the MAPP ML cluster affiliated with each body map phenotype

We next examined how the ML-derived MAPP clusters aligned with the distribution of body pain sites in the body pain inventory. This cohort has previously been grouped into body-map (BM) phenotypes of widespread pain, intermediate pain, and pelvic pain only (PPO). While the differences were more pronounced when participants were separated exclusively on this criterion, the MAPP ML clusters exhibited a similar pattern in the number of painful sites outside the pelvis as the BM phenotypes (Fig. 4A). C2-NUPP had the most confined pain, similar to PPO, with the fewest pain sites outside the pelvis. C1-MFP endorsed the most sites of pain outside the pelvis, like the widespread pain group, and the C3-BPS cluster had an intermediate phenotype. There was only partial overlap, however, between ML cluster identity and BM phenotype assignment (Fig. 5). For example, even though many C2-NUPP cluster participants were also assigned to the PPO BM phenotype, a significant number (over half) were actually assigned to other phenotypes. Thus, despite similar patterns in pain distribution, there was poor consistency between the two classification systems. Unlike the ML cluster classification, the BM phenotypes did not display any significant differences in urinary, painful, or QoL indices on other symptomatic measures (e.g., fGUPI, ICPI, ICSI) (Fig. 4C–E).

## Discussion

We recently proposed a simple phenotyping schema for IC/BPS using unsupervised ML, circumventing the need for complex clinical profiling or detailed exams [16]. Classifying patients using patient-reported symptomatic measures alone, we described three phenotypes (MFP, NUPP, BPS) with distinct pain and urinary symptomatic patterns and differential responses to IC/BPS therapies. In this report, we reproduce these findings in a multi-center, prospectively recruited national IC/BPS cohort. In the SC cohort, cluster definitions were generated using five symptomatic questionnaires including 49 individual features. We independently derived the three phenotypes in the MAPP cohort using only 11 features (fGUPI subscales and ICSI/ICPI items). Despite fewer features, unsupervised ML classified this independent, multisite cohort into similar symptom clusters with equivalent urinary and pain symptom patterns and physical exam findings as the SC cohort. The robust nature of these clusters across cohorts suggests these differences likely reflect true clinical and pathophysiologic differences.

While similar to the SC clusters in symptomatic patterns, the MAPP clusters overall presented with higher symptomatic severity (Fig. 3), likely reflecting differences in recruitment. The SC cohort was a cross-sectional population requiring only a single visit for questionnaire assessment, while the MAPP was a year-long study requiring multiple visits, extensive questionnaires, and biological sampling. It is reasonable to assume this latter cohort would comprise participants more severely affected by their symptoms, who were more willing to participate in longitudinal evaluation. Despite these population differences, ML clustering exhibited good reliability and reproducibility in both populations; the classifier consistently detected distinct symptom patterns, not differences in symptom severity. The Rand indices supported good percent observed agreement of clusters. The Jaccard indices, however, demonstrated suboptimal overlap between bootstrapped clusters, recognizing how similar these clusters are in symptomatic manifestations. Clusters share elevated scores for most symptomatic measures; these phenotypes cannot be identified based on single pathognomonic features, requiring recognition of different patterns combining several prominent features. Regardless of these similarities, unsupervised clustering allowed for reliable discrimination of phenotypes from a limited set of patient-reported symptoms across a wide range of subject types in diverse geographical regions. While additional studies will need to validate the ability of this classification approach to improve treatment assignments, as suggested by our preliminary studies [16], these early results show promise for clinical utility in a range of practice environments.

Recently, the MAPP research network categorized IC/BPS patients by pain distribution (widespread, intermediate, and local pelvic pain) directed by the clinical observation that patients with widespread pain behave differently than those with local pelvic pain. If widespread pain represents a pathophysiology distinct from localized pain, we would expect differences in symptomatic patterns. However, the body mapping based phenotypes revealed minimal symptomatic differences between groups. While the ML clusters described in this study demonstrated poor overlap with these body map clusters, the underlying concept noting differences in widespreadness of pain persisted in the ML model. The C1-MFP group demonstrated widespread pain, with significant extrapelvic pain sites and the most severe overall painful complaints unrelated to urologic or pelvic pain. The C2-NUPP group had the most confined pain while the C3-BPS cluster defined an intermediate phenotype suggesting that while widespreadness of pain may reflect differences in IC/BPS clinical course, solely relying on body mapping for phenotypic classifications may not adequately capture the underlying pathophysiologies driving these patterns.

Consistent with this hypothesis, a separate study subdivided the MAPP cohort according to pelvic floor tenderness, noting that participants with more severe pelvic floor tenderness exhibited more severe symptoms, a wider distribution of non-urologic pain, and worse QoL [21]. This group is highly analogous to the C1-MFP group. Despite disparate methods of identification, one using physical exam findings and the other utilizing patient-reported symptoms, the phenotypes are highly similar, supporting the hypothesis that the MFP phenotype represents a physiologically and clinically different pathology from the BPS subgroup.

Recognizing myofascial pain in pelvic pain cases typically relies on a discriminate pelvic exam conducted by a provider experienced in identifying myofascial dysfunction [22, 23] which can pose a challenge for many physicians, especially non-specialists. Furthermore, in the era of increasing virtualization, patients may not always have the opportunity for in-person care. By distinguishing IC/BPS phenotypes using patient reported symptoms, this approach can provide assistance in this initial evaluation, especially in the context of telemedicine or for providers who may not feel comfortable assessing the pelvic floor. The ML classifier offers a potential solution to these obstacles using patient-reported symptom-based classification, potentially enhancing the recognition of these different phenotypes, even when providers are unfamiliar with comprehensive pelvic pain assessment and supporting physical exam.

While subtle, the cluster phenotypes exhibit several informative differences in pain patterns. The NUPP cluster stands out as less severe overall, exhibiting pain unrelated to the bladder cycle; only pain localized to the vagina and introitus, exacerbated by intercourse, was similar to the other groups. Distinction of the BPS and MFP phenotypes, however, is challenging. Both groups share high levels of bladder pain, severe urge and frequency, and poor QoL. However, unsupervised clustering reveals critical distinctions between the phenotypes consistent with different etiologies. The BPS cluster experiences relief with emptying the bladder, suggesting that distention/perturbation of the bladder is involved in the generation of pain, consistent with the documented efficacy of bladder-directed therapies in this population. In contrast, the MFP group experiences persistent discomfort regardless of bladder volume, suggesting an etiology extrinsic to the bladder.

Historically, MFP was hypothesized to be a *consequence* of bladder-specific pain due to pelvic nerve sensitization, as patients perceived their pain to be originating from the bladder [24]. However, the perceived bladder pain in the MFP phenotype may represent *referred pain* originating outside the bladder, possibly instigated by the pelvic floor musculature [25, 26]. A similar phenomenon is seen in rectalgia; patients perceive pain in the rectum, but the culprit is levator ani muscle spasm [27, 28]. While these findings do not dismiss the possibility of pelvic floor muscle dysfunction in BPS patients, accumulating evidence suggests that the MFP phenotype represents a distinct, frequently unrecognized subset of IC/BPS. Coupled with the high response rates to pelvic floor physical therapy [16, 29], these data support the hypothesis that bladder pain in the MFP phenotype is primarily driven by myofascial dysfunction rather than being a consequence of bladder pain.

Given the widespread pain in the MFP phenotype, some of these patients may have a centralized pain pathology contributing to muscular hypertonicity/pain, with bladder pain being a prominent symptom. IC/BPS often co-exists with other somatic syndromes such as fibromyalgia, IBS, and psychiatric comorbidities (e.g., anxiety, trauma, and depression) [30], many of which exhibit increased sympathetic output, muscular trigger points, and hypertonicity. Future studies will need to examine whether the MFP phenotype is selectively associated with these comorbidities.

This study is limited to the examination of younger women, to isolate phenotypes without confounders related to menopause and other age-related conditions. In addition, we excluded women on chronic opioids, to avoid possible blunting of painful symptoms critical in distinguishing phenotypes. Additional studies will need to determine the applicability of this classification system to other populations (e.g., men, older women). Additionally, both SC and MAPP individuals were identified and evaluated by specialist physicians experienced in the evaluation of pelvic pain. It is unclear how the model would perform in a broader population of pelvic pain patients; additional symptomatic features may be needed to distinguish urologic pain from other pelvic pain groups (e.g., endometriosis). Our study was cross-sectional in nature; we did not evaluate the stability, evolution, or treatment responses of these clusters over time. As treatments were not prospectively assigned, the wide variety of possible treatments and large number of pre-existing treatments at enrollment makes it challenging to attribute treatment effects to specific clusters. Additional studies will be necessary to determine if ML clustering can predict treatment responses. Lastly, diagnostic testing results (such as urodynamic studies, imaging, cystoscopy) were not evaluated in this study. Nevertheless, it is encouraging that consistent IC/BPS phenotypes were identified using patient-reported symptom questionnaires alone in both the SC and MAPP cohorts.

In conclusion, patient-reported symptoms can be utilized to identify distinct subgroups of perceived bladder pain that previously required discriminate pelvic exam and detailed clinical history to differentiate. The characteristic symptomatic patterns were reproducible between a single institution cohort and a multi-center population, recapitulating previous findings relating clinical outcomes to differences in widespreadness of pain and pelvic floor muscle tenderness. Further validation of these phenotypes in additional populations and determination of their associations with response to therapy will further solidify our collective understanding of the distinct underlying pathophysiologies that underpin bladder pain.

### Supplementary Information

Below is the link to the electronic supplementary material.

Supplementary file1 (DOCX 15 KB)

Supplementary file2 (DOCX 13 KB)

## Data Availability

Data is available upon request from the corresponding author, Dr. A. Lenore Ackerman.

## Abbreviations

MAPP: Multidisciplinary approach to the study of chronic pelvic pain
IC/BPS: Interstitial cystitis/bladder pain syndrome
UCPPS: Urologic chronic pelvic pain symdrome
ML: Machine learning
MFP: Myofascial pelvic pain
NUPP: Non-urologic pelvic pain
BPS: Bladder-specific pain
SC: Single center
fGUPI: Female genitourinary pain index
ICSI: Interstitial cystitis symptom index
ICPI: Interstitial cystitis problem index
AUA-SI: American Urological Association Symptom Index
RICE: RAND Interstitial Cystitis Epidemiology Study Case Definition Questionnaire
SymQ: Symptom and Health Care Utilization Questionnaire
BM: Body map
PPO: Pelvic pain only
QoL: Quality of life
