# Supplementary material for: Validation of Distinct Bladder Pain Phenotypes Utilizing the MAPP Research Network Cohort
Source: Int Urogynecol J. 2024 Feb 1;35(3):637–48. doi: 10.1007/s00192-024-05735-1 (PMC11023803; doi:10.1007/s00192-024-05735-1)
Supplement: Supplementary file 1 — Supplementary file1 (DOCX 15 KB) [file 192_2024_5735_MOESM1_ESM.docx]

| **Cluster** | **MAPP C1-MFP** | **MAPP C3-BPS** | **MAPP-C2-NUPP** | **p** |
| --- | --- | --- | --- | --- |
| **n** | **(31)** | **(52)** | **(47)** |  |
| **Female Genitourinary Pain Index (fGUPI)**  Pain at Introitus (fGUPI1a, n (%)) | 16 (51.6) | 22 (44.9) | 16 (34.0) | 0.280 |
| Pain In Vagina (fGUPI1b, n (%)) | 17 (54.8) | 24 (47.1) | 18 (38.3) | 0.347 |
| Pain in Urethra (fGUPI1c, n (%)) | 20 (64.5) | 28 (53.8) | 14 (29.8) | 0.006 |
| Pain Below the Waist (fGUPI1d, n (%)) | 30 (96.8) | 49 (94.2) | 40 (85.1) | 0.129 |
| Pain with Urination (fGUPI2a, n (%)) | 25 (80.6) | 26 (50.0) | 13 (27.7) | <0.001 |
| Pain with Intercourse (fGUPI2b, n (%)) | 18 (60.0) | 29 (59.2) | 21 (44.7) | 0.271 |
| Pain with Bladder Filling (fGUPI2c, n (%)) | 26 (83.9) | 47 (90.4) | 19 (40.4) | <0.001 |
| Pain relieved by Bladder Emptying (fGUPI2d, n (%)) | 20 (64.5) | 38 (73.1) | 23 (48.9) | 0.045 |
| Frequency of Pain (fGUPI3, mean (SD)) | 4.13 (1.06) | 3.38 (1.14) | 2.49 (1.20) | <0.001 |
| Average Pain or Discomfort (fGUPI4, mean (SD)) | 7.16 (1.51) | 5.77 (1.64) | 3.94 (1.89) | <0.001 |
| *fGUPI Pain Subscale (mean (SD))* | 16.93 (2.86) | 14.26 (3.23) | 9.91 (3.80) | <0.001 |
| Sensation of Incomplete Emptying (fGUPI5, mean (SD)) | 5.65 (0.75) | 4.92 (1.20) | 2.81 (1.19) | <0.001 |
| Urinary Frequency (fGUPI6, mean (SD)) | 4.87 (1.28) | 4.15 (1.56) | 2.23 (1.32) | <0.001 |
| *fGUPI Urinary Subscale (mean (SD))* | 8.52 (1.73) | 7.08 (2.17) | 3.04 (1.84) | <0.001 |
| Symptom Interference (fGUPIq7, mean (SD)) | 2.26 (0.86) | 1.65 (0.93) | 0.79 (0.86) | <0.001 |
| Distraction (fGUPIq8, mean (SD)) | 2.84 (0.45) | 2.56 (0.67) | 1.83 (0.82) | <0.001 |
| Symptom Satisfaction (fGUPIq9, mean (SD)) | 5.29 (1.01) | 4.77 (1.15) | 3.32 (1.40) | <0.001 |
| *fGUPI QOL Subscale (mean (SD))* | 10.39 (1.93) | 8.98 (2.28) | 5.94 (2.57) | <0.001 |
| ***Total GUPI Score (mean (SD))*** | 35.83 (4.86) | 30.32 (5.06) | 18.89 (5.91) | <0.001 |
| **Interstitial Cystitis Symptom Index (ICSI)**  Urinary Urgency (ICSIq1, mean (SD)) | 3.81 (1.17) | 1.96 (1.37) | 1.13 (1.19) | <0.001 |
| Urinary Frequency (ICSIq2, mean (SD)) | 4.77 (0.56) | 3.96 (1.12) | 1.85 (1.06) | <0.001 |
| Nocturia (ICSIq3, mean (SD)) | 3.61 (1.09) | 1.85 (1.04) | 1.43 (1.12) | <0.001 |
| Bladder Pain/Burning (ICSIq4, mean (SD)) | 3.15 (1.36) | 4.13 (1.12) | 2.09 (1.50) | <0.001 |
| ***ICSI Total Score (mean (SD))*** | 16.32 (1.62) | 10.92 (2.04) | 6.49 (2.38) | <0.001 |
| **Interstitial Cystitis Problem Index (ICPI)**  Urinary Frequency (ICPIq1, mean(SD)) | 3.61 (0.50) | 2.94 (0.85) | 1.19 (0.97) | <0.001 |
| Nocturia (ICPIq2, mean (SD)) | 3.65 (0.55) | 2.33 (1.18) | 1.28 (1.12) | <0.001 |
| Urinary Urgency (ICPIq3, mean (SD)) | 3.03 (0.91) | 1.56 (1.19) | 0.96 (1.20) | <0.001 |
| Bladder Burning/Pressure (ICPIq4, mean (SD)) | 3.35 (0.84) | 3.81 (0.40) | 2.19 (1.06) | <0.001 |
| ***ICPI Total Score (mean(SD))*** | 14.10 (1.42) | 10.17 (2.22) | 5.62 (2.40) | <0.001 |

**Supplemental Table 1*. Scores on clustering features used to generate of MAPP ML clusters.*** Geometric mean scores and standard deviations (mean (SD)) or total numbers of subjects endorsing symptoms with percentages affected (n (%)) are shown as indicated for pain and urinary symptomatic measures. fGUPI, female Genitourinary Pain Index; ICSI, Interstitial Cystitis Symptom Index; ICPI, Interstitial Cystitis Problem Index.
