# Supplementary material for: Validation of Distinct Bladder Pain Phenotypes Utilizing the MAPP Research Network Cohort
Source: Int Urogynecol J. 2024 Feb 1;35(3):637–48. doi: 10.1007/s00192-024-05735-1 (PMC11023803; doi:10.1007/s00192-024-05735-1)
Supplement: Supplementary file 2 — Supplementary file2 (DOCX 13 KB) [file 192_2024_5735_MOESM2_ESM.docx]

|  | Observations | Mean | Std. Dev. |
| --- | --- | --- | --- |
| Rand Index  MAPP C1-MFP  MAPP C2-NUPP  MAPP C3-BPS | 500  500  500 | 0.76  0.76  0.74 | 0.21  0.34  0.12 |
| Jaccard Coefficient  MAPP C1-MFP  MAPP C2-NUPP  MAPP C3-BPS | 500  500  500 | 0.51  0.64  0.42 | 0.29  0.34  0.28 |

**Supplemental Table 2. *Cluster Stability Indices for MAPP ML clusters.***
